# Occlusal Contact Changes in Implant‐Supported Fixed Prostheses: A Systematic Review

**DOI:** 10.1111/joor.70015

**Published:** 2025-07-24

**Authors:** Itt Assoratgoon, Ramadhan Hardani Putra, Hiroki Hihara, Tetsuo Kawata, Takahiro Chiba, Pimduem Rungsiyakull, Nobuhiro Yoda

**Affiliations:** ^1^ Division of Advanced Prosthetic Dentistry Tohoku University Graduate School of Dentistry Sendai Japan; ^2^ Geriatric Dentistry and Special Patients Care Program Faculty of Dentistry, Chulalongkorn University Bangkok Thailand; ^3^ Department of Dentomaxillofacial Radiology Faculty of Dental Medicine, Universitas Airlangga Surabaya Indonesia; ^4^ Department of Prosthodontics Faculty of Dentistry, Chiang Mai University Chiang Mai Thailand

**Keywords:** implant‐supported crowns, implant‐supported prostheses, occlusal contact area, occlusal contact changes, occlusal force, occlusal time

## Abstract

**Background:**

Implant‐supported prostheses frequently face both mechanical and biological challenges. Although various techniques and principles are employed to manage excessive loads, occlusal contacts often change within 6 months of placement.

**Objective:**

This review aims to analyse relevant studies to evaluate the changes in occlusal contacts that occur following the placement of superstructures in implant‐supported prostheses.

**Methods:**

The protocol was designed according to the Preferred Reporting Items for Systematic Reviews and Meta‐Analyses Protocols (PRISMA‐P) and registered with the International Prospective Register of Systematic Reviews. Studies were included if their outcomes addressed stress distribution, occlusal force, occlusal contact, occlusal scheme or changes in occlusal time. Non‐English articles, case reports, animal experiments, systematic reviews and literature reviews were excluded.

**Results:**

A total of 1867 articles were retrieved through database searches, along with an additional 43 articles identified through manual searches, of which 416 were duplicates. After screening the titles and abstracts, 1367 articles were excluded, and 12 were deemed non‐retrievable. The full texts of the remaining 85 articles were independently reviewed by two authors for eligibility, resulting in the exclusion of 77 articles based on the inclusion criteria. Ultimately, eight articles were included in the review.

**Conclusion:**

Implant‐supported crowns gradually approach or surpass the load of natural teeth over time, as evidenced by increased occlusal force, larger contact areas and longer occlusion durations. These findings highlight the need for more accurate and advanced measurement techniques to better understand and manage the long‐term changes in occlusion associated with implant‐supported crowns.

**Trial Registration:**

PROSPERO: CRD42024527043

## Graphical abstract

Overview of this research.

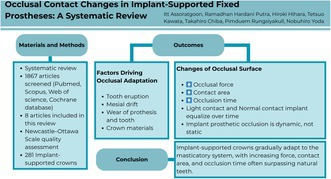

## 1 Introduction

Implant‐supported prostheses have been a cornerstone of prosthetic dentistry for decades. However, complications are undeniably associated with implant‐supported prostheses, arising from both mechanical and biological factors [1, 2]. One fundamental difference between implant‐supported and natural dentition lies in the absence of periodontal ligaments in implants, which can lead to an overload on the implant and surrounding bone, potentially causing mechanical and biological complications such as microfractures at the bone–implant interface and within the bone tissue itself [1, 2, 3]. The lack of periodontal ligaments limits the displacement of implant‐supported prostheses under an applied occlusal force, with vertical and lateral movements being significantly reduced compared to natural teeth. Natural teeth exhibit vertical movements ranging from 25 to 100 μm and lateral movements from 56 to 108 μm. In contrast, implants display a range of only 3–5 μm for vertical movements and 10–50 μm for lateral movements [1, 4].

The biophysical differences between natural teeth and dental implants may prevent dentists from applying natural teeth occlusion concepts to implants [5]. Consequently, the concept of implant‐protected occlusion (IPO) was developed to mitigate the load on implants and peri‐implant bone by constructing proper occlusion of the superstructure on the implants opposing the natural teeth [6, 7, 8]. IPO recommends that implant occlusion should avoid heavy contact, permitting only light occlusion during minimal contact, ideally maintaining a 30‐μm gap between the surfaces [9]. Maintaining stable occlusion after superstructure placement can significantly reduce mechanical and biological complications, thereby increasing prosthesis longevity [10].

Despite efforts to minimise implant loads, light occlusal contact achieved at the time of placement often does not remain consistent throughout the implant's lifespan. Changes in occlusal contact commonly occur within the first 6 months of superstructure placement [11, 12, 13, 14]. Furthermore, assessing the surface topography of superstructures and the force applied to the implant remains challenging owing to limitations in conventional methods. Techniques involving articulating paper, shim‐stock film and diagnostic casts have been questioned for their reliability in quantifying occlusal changes [15, 16]. Understanding these changes and their causes is crucial, as the occlusal contact area is closely related to the occlusal force impacting the implant and surrounding bone.

This systematic review aimed to analyse relevant studies and evaluate the changes in occlusal contacts that occur following the placement of the superstructure in the implant‐supported prosthesis.

## 2 Materials and Methods

### 2.1 Protocol and Registration

**FIGURE 1:**
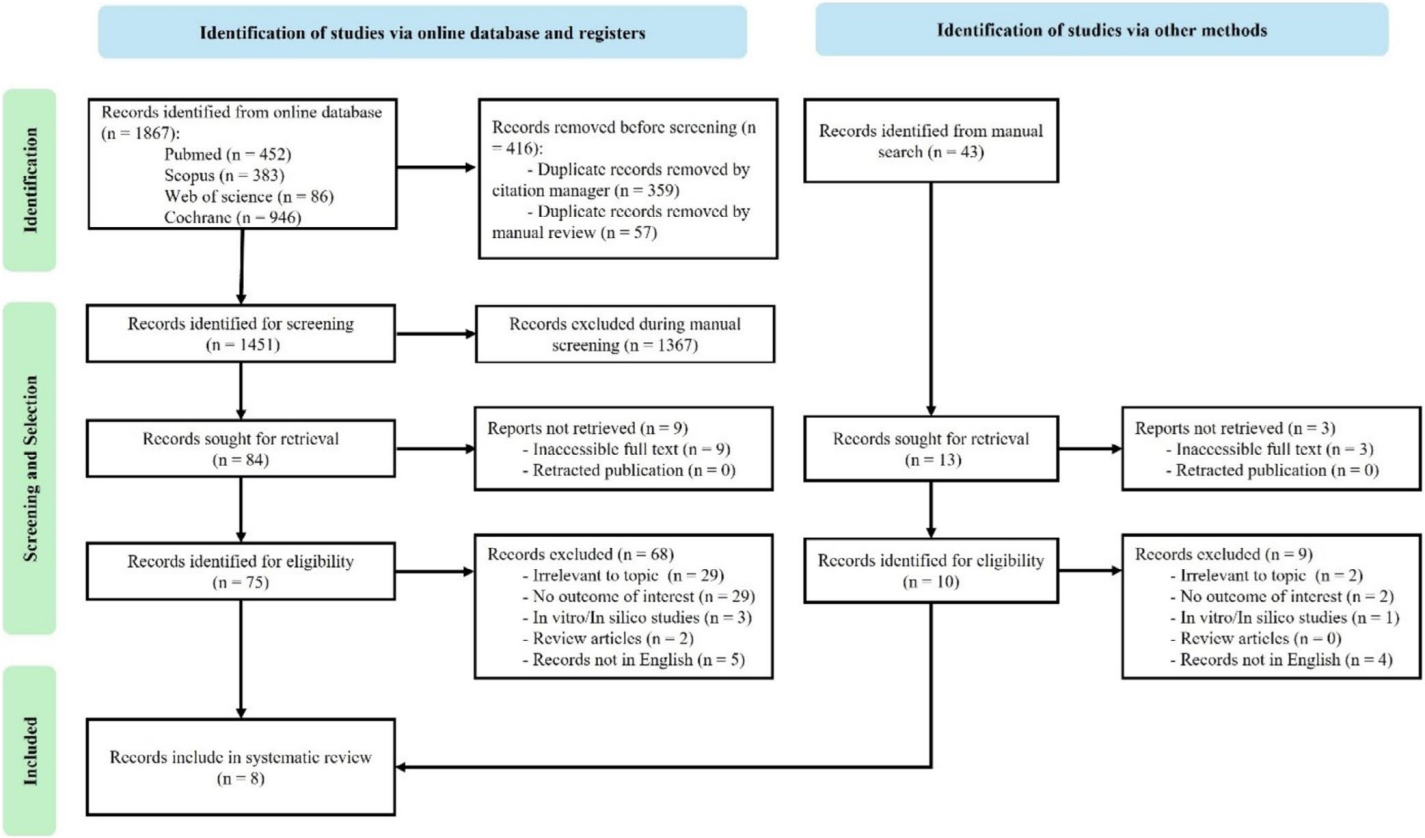
PRISMA flow diagram.

This review was conducted in accordance with the Preferred Reporting Items for Systematic Review and Meta‐Analyses (PRISMA) statement (Figure 1). The review protocol was registered in the International Prospective Register of Systematic Reviews.

### 2.2 Eligibility Criteria

The search strategy was based on the PECO framework as follows: population, patients with implant‐supported single crowns; exposure, superstructure placement; comparator: occlusal surface before superstructure placement; outcome, occlusal contact changes.

The inclusion criteria were as follows: prospective studies on occlusal contact surface changes in a single implant, a minimum of 10 participants, outcomes related to stress distribution, occlusal force, occlusal contact, occlusal scheme, occlusal time change or other measures evaluating post‐superstructure occlusion differences. Articles were excluded for the following reasons: non‐English languages, case reports, review articles, studies involving patients with uncontrolled systemic diseases or local defects and animal experiments.

### 2.3 Information Sources

Online database searches were conducted in the PubMed, Scopus, Web of Science and Cochrane databases from inception until 31 October 2024. Manual searches of reference lists from related articles, systematic reviews and meta‐analyses were also performed to supplement the database search.

### 2.4 Article Selection

Article selection was independently performed by two trained reviewers (I.A. and H.H.) in a two‐phase process: screening titles/abstracts and reviewing full texts. Disagreements at either phase were resolved through discussion until mutual agreement was achieved. Discrepancies were resolved through a consensus session with the study coordinator (N.Y.).

### 2.5 Data Collection Process

Data extraction from the selected articles were independently conducted by two researchers (I.A. and H.H.). The data collected included the study information (author, year of publication, study design and country), population details (sample size, sex, age and site of study), intervention (type of prosthesis, dental arch, number and characteristics of implants, groups of comparison and follow‐up time) and outcomes (dropouts, occlusal contact number, occlusal contact area, occlusal force, mastication parameters and main findings).

### 2.6 Quality Assessment

The study quality was independently assessed by two reviewers (I.A. and H.H.) (Table 1). All the included studies were cohort studies, and the Newcastle–Ottawa Scale was used to evaluate the quality of the included studies. Quality assessment was based on three aspects: (1) selection, (2) comparability and (3) outcomes. A maximum of nine stars could be awarded to each study: four for the selection of study groups, two for the comparability of the groups and three for the ascertainment of either exposure or outcomes of interest. The studies were rated as follows: good quality: three or four stars in the selection domain, one or two stars in the comparability domain and two or three stars in the outcome/exposure domain; fair quality: two stars in the selection domain, one or two stars in the comparability domain and two or three stars in the outcome/exposure domain; and poor quality: zero or one star in the selection domain, zero stars in the comparability domain or zero or one star in the outcome/exposure domain [25].

**TABLE 1:** Article quality assessment using the Newcastle–Ottawa Scale.

Article	Selection				Comparability		Outcome			Total (9/9)	Rating
	Representative of the exposed cohort	Selection of the external control	Ascertainment of exposure	Outcome of interest at start of study	Main factor	Additional factor	Assessment of outcomes	Sufficient follow‐up time	Adequacy of follow‐up		
M. N. Madani, 2017 [17]	*	0	*	*	*	0	*	*	*	(7/9)	Good
K. Kon, 2017 [18]	*	*	*	0	*	*	*	*	*	(8/9)	Good
L. Qiang, 2020 [19]	*	0	*	0	*	*	*	*	0	(6/9)	Fair
Y. J. Kim, 2021 [20]	*	0	*	*	*	*	*	*	*	(8/9)	Good
T. Zhou, 2021 [21]	*	0	*	*	*	0	*	*	0	(6/9)	Good
Q. Ding, 2022 [22]	*	0	*	0	*	0	*	*	0	(5/9)	Fair
R. Zhang, 2023 [23]	*	0	*	0	*	*	*	*	*	(7/9)	Fair
Y. Zhang, 2023 [24]	0	0	*	*	0	*	*	*	*	(6/9)	Fair

*Note:* *indicates that the item is applicable.

## 3 Results

### 3.1 Literature Search

A total of 1867 articles were identified through keyword searches. A total of 416 duplicated articles were removed using a reference management programme (EndNote 21, Clarivate Analytics, UK) and manual detection, and 1367 articles were excluded during the title and abstract screening process. This narrowed the number of eligible articles to 84. The full texts of nine articles could not be retrieved, and 68 were excluded for not meeting the inclusion criteria. Seven articles were included in the final analysis from the online database search and one from manual searches of the reference lists of related articles. Finally, eight articles fulfilled the inclusion criteria and were included in this review (Table 2) [17, 18, 19, 20, 21, 22, 23, 24].

**TABLE 2:** Characteristics of included articles.

Year	First author	Number of patients	Sample size (crown)	Age (year)	Crown material	Crown location	Outcome measurement	Main conclusion
2017	M. N. Azam S Madani [17]	21 (10F/10M)	21	18–48 (30.81 ± 8.85)	Metal‐ceramic	Maxilla and mandibule Premolar (13) Molar (8)	Percentage of applied force per tooth (of the implant‐supported crown and the opposite tooth)	Percentage of force of the implant‐supported tooth became higher at 3 and 6 months. Percentage of force of the natural tooth became lower at 3 and 6 months.
2017	K. Kon [18]	14 (9F/5M)	21	41–72 (61.4)	Metal‐ceramic	Maxilla and mandibule Premolar (0) Molar (21)	Occlusal force, occlusal area	Free end missing cases shows increased occlusal force and occlusal area on the side of the implant. Reduced force and area for other natural teeth on the implant side.
2020	L. Qing [19]	33 (18F/15M)	37	23.9–70 (42.8)	Ceramic, metal‐ceramic, metal‐resin, metal crown	Maxilla and mandibule Premolar (7) Molar (30)	Occlusal force, occlusal time	Occlusal force of implant increased to match control teeth after 3 months and remained similar afterward. Occlusal time ratio increased between 0.5–3 months and 3–6 months. Significant correlation between occlusal force and occlusal time ratio at 0.5, 3, and 6 months.
2021	Y. J. Kim [20]	50 (19F/31M)	50	38–78	Gold, Co‐Cr	Maxilla and mandibule Premolar (0) Molar (50)	Occlusal force, occlusal contact area	Total occlusal force increased after 1 month (first molar showed a more significant change than second molar). Contact area of the implant increased when measuring with a 9‐μm‐thick articulating paper after 1 month. Contact area of the mesial teeth increased after 1 month.
2021	T. Zhou [21]	30 (18F/12M)	32	27–75	Zirconia	Maxilla and mandibule Premolar (N/A) Molar (N/A)	Bite force distribution, bite force deviation	Occlusal force of the implant crown significantly increased during the 3‐month follow‐up.
2022	D. Qing [22]	33 (17F/16M)	37	23.9–70	Metal‐ceramic Metal‐resin Metal	Maxilla and mandibule Premolar (7) Molar (30)	Occlusal force, occlusal time	Occlusal force of the implant‐supported crowns increased between 0.5 and 36 months. Not significant afterward. Occlusion time increased between 0.5 and 6 months. Not significant afterward. Occlusal force compared with the control increased until it exceeded control at 48 and 60 months.
2023	R. Zhang [23]	50 (27F/23M)	50	36.97 ± 7.34	Zirconia	Maxilla and mandibule Premolar (0) Molar (50)	Occlusal force, occlusal time, asymmetry index of occlusal force, visual analog scale of satisfaction	Relative occlusal force of both groups increased to exceed that in the control group eventually. Light contact group took longer to achieve the same value as that in the control. Occlusal contact time of both groups decreased over time, with the light contact group being later than the normal group. The difference gradually decreased.
2023	Y. Zhang [24]	33 (12F/21M)	33	25–69 (46.8)	LS_2_, ZrO_2_	Maxilla and mandibule Premolar (8) Molar (25)	Occlusal clearance, occlusal contact area	Occlusal clearance decreased. Occlusal contact increased.

### 3.2 Patient Demographic

Among the included articles, the ages of patients ranged from 16 to 78 years. Four articles reported the mean patient age, which ranged from 30.8 to 61.4 years [17, 18, 19, 23, 24]. The total number of patients in each study ranged from 10 to 50, and the number of implants studied varied from 12 to 50. All implants were located exclusively in the posterior region both in the maxilla and mandible [17, 18, 19, 20, 21, 22, 23, 24].

### 3.3 Loading Protocol

Two studies did not report the loading protocols for the included prostheses [21, 24]. The remaining articles required a minimum healing period of 3 months following implant placement before superstructure placement. Of these, three studies reported that the superstructure was placed 4–5 months after implantation [19, 22, 23]. One study reported a healing time of 3 months [18], while another specified a healing period of 3 months for mandibular cases and 6 months for maxillary cases [20].

### 3.4 Study Outcomes

The outcomes assessed were occlusal force [18, 19, 20, 21, 22, 23], occlusal time [19, 22, 23], occlusal contact area [18, 20, 24], bite force distribution and deviation [21], occlusal clearance [24] and visual analog scale of patient satisfaction [23].

### 3.5 Quality Assessment

The quality of the articles was assessed using the Newcastle–Ottawa Scale, and the scores are summarised in Table 1. Of the total studies, four were of good quality, and four were of fair quality. The mean quality assessment score of the included studies was 6.63.

## 4 Discussion

### 4.1 Consideration of Implant Prosthesis Occlusion

The occlusion of implant prostheses is a critical concern owing to the potential risk of clinical failures, such as damage to implant components or surrounding tissues caused by inappropriate loading scenarios. This risk may increase further over time as the superstructure continues to function [26]. The underlying cause of these occlusion‐related problems lies in the fundamental differences between osseointegrated dental implants and natural teeth with periodontal apparatuses. Specifically, without the support of a periodontal structure, implants lack the tactile sensibility and mobility necessary to compensate for loading [27, 28, 29]. Consequently, the proprioception of implant‐supported prostheses is reportedly significantly lower than that of natural teeth [29]. To address this challenge, the concept of IPO was developed. This approach aims to reduce the force at the crestal bone–implant interface by guiding treatment planning with biomechanical principles. These principles include modifying the occlusal table and contact, adjusting the direction of forces and eliminating force magnifiers, such as cantilevers and offset loads [8]. The impaired occlusal sensation of the implant can be compensated for by increased sensation of muscle spindles, sensory receptors in the temporomandibular joint, osseoperception or enhanced periodontal sensation of the opposing tooth [4]. Recovery of these peripheral feedback pathways can be considered a physiological adaptation of the implant to the stomatognathic functional system. However, this adaptation may require time and rehabilitation for smooth oral function [30]. Importantly, the accuracy of occlusal sensation immediately after superstructure attachment and the recovery process varies among individuals.

### 4.2 Measurement Methods

The primary challenge in the included studies was the measurement methods used to assess these occlusal changes, including factors such as occlusal force, occlusal time and contact area. However, determining the most suitable outcome for assessing these changes is essential. The included studies used various measurement techniques to evaluate changes; however, none compared the outcomes in terms of their effects on the implants or prostheses. One study examined the relationship between the relative occlusal force (defined as the percentage of the prosthesis of interest against the total occlusal force) and the mesial and distal marginal bone levels of the implant. The results showed a positive relationship, indicating that increased relative force correlated with higher marginal bone levels [22]. However, the author noted that other potential factors limit the clinical relevance of this finding. Another study assessed patient satisfaction using visual analog scales and found that scores increased with greater occlusal force, likely because of improved mastication efficiency [23].

The most commonly used device for measuring occlusal force among the included studies was the T‐Scan sensor system (Tekscan) [19, 21, 22, 23], a computerised occlusal force analysis device that displays measurable bite force data in both 2D and 3D for every area of the dentition [31]. Two studies used a pressure‐sensitive film and a scanner system (Dental Prescale, Fuji Film Corp., Japan) [18, 20]. Notably, the force measured using both devices was the maximum bite force at each time point, which may not fully represent the daily functional force. Furthermore, neither could assess the direction of the force. Although pressure‐sensitive films and scanner systems are easier to use, the thin sheet between the occlusal surfaces limits their ability to replicate natural chewing and biting motions. This contrasts with more advanced bite force–measuring devices, such as piezoelectric and strain‐gauge transducers. However, these advanced approaches are less feasible for large patient studies because of their high costs and time requirements [32].

Occlusal contact time on the implant‐supported crown, defined as the time from first occlusal contact to the maximum intercuspal position, was measured using a digital occlusion analysis system [19, 22, 23]. Of the three studies that investigated occlusal contact time, two used the ratio of the occlusal contact time of the crown‐supported implant to the occlusal time of the entire dentition [19, 22], whereas one measured the direct value of the occlusal contact time for the implant of interest [23]. The occlusal contact area was assessed using data from the T‐Scan and silicone impression of the dentition [18, 20]. Zhang et al. [24] used 3D models of restorations to calculate contact area using image analysis software (Geomagic 2015, 3D Systems).

Overall, the consensus among the studies is that conventional techniques for evaluating occlusal contacts, such as those using articulation paper, diagnostic casts, and shim‐stock foils, are inadequate for precisely measuring occlusal changes. Moreover, using articulating papers is not advisable for assessing load intensity on the crown, as no correlation exists between the size of the marks and the magnitude of the force [31, 33, 34]. The optimal approach involves adopting digital dentistry technologies, such as intraoral scanners and force‐measuring methods that avoid placing materials between the occlusal surfaces to better replicate the natural jaw function.

### 4.3 Occlusal Changes in Implant‐Supported Prothesis

A single implant‐supported crown is commonly designed to have a smaller contact area than that of a natural tooth. This helps alleviate the burden on the implant, especially during the initial stages of healing and osteointegration [8, 27, 35]. However, these results suggest that this light contact state is not stable over time [20, 22, 23]. The consensus among the included studies was that, over time, the functionality of the implant‐supported crown within the masticatory system improves. This may be related to the increased load exerted on the crown, larger contact area and prolonged occlusion time [18, 19, 20, 21, 22, 23].

Initially, the implant‐supported crown may not bear the same intensity of force as adjacent natural teeth. However, over time, the load it receives approaches, and in some cases, even exceeds that of the natural teeth [22, 23]. In a study comparing normal and light contact implants at the time of superstructure placement, the results showed that the occlusal force in both groups eventually exceeded that of natural teeth, although the light contact group required more time to reach this level [23].

In addition to altering the occlusion of the implant‐supported crown, these changes can also affect other parts of the dentition. The difference between implants located at the free end of the arch and those placed between natural teeth is that implants at the free end cause changes in the contact area and loading force across the entire side of the dental arch, unlike implants positioned between natural teeth. This finding suggests that placing an implant at the free end may facilitate a more balanced distribution of force across the overall occlusion [18].

### 4.4 Factors Contributing to Occlusal Changes

Implants lacking a periodontal ligament can exert higher occlusal forces and make contact earlier than adjacent natural teeth, especially if a slight infraocclusion is intended at the time of superstructure placement. However, that condition may not be maintained, as discussed earlier, and tends to diminish over time [24, 29].

Even in cases where the vertical dimension of occlusion is restored through prosthetic treatment, subsequent changes may occur, which often stabilise within 1 year [36]. In cases where mandibular deviation has been restored through pretreatment, it is essential to allow for a sufficient period of provisional restoration to assess the stability of both the mandibular position and occlusion before proceeding with the final prosthetic treatment.

After osseointegration, the implant became stationary, and the continuous eruption process ceased. In contrast, the opposing natural tooth may continue erupting in response to the light contact made at the time of superstructure delivery and the wear of both the natural teeth and superstructure. Moreover, the 0.1–0.2‐mm mesial movement of the opposing teeth contributes to the change [11]. This continuous eruption may have a greater impact than the wear of both the opposing tooth and prosthesis on altering the occlusion [19].

Zhang et al. tested the change in the occlusal contact area between monolithic lithium disilicate and zirconia crowns. The results indicated an increase in the contact area for both materials; however, the difference in the changes between the two materials was not significant [24].

Another factor not addressed by the authors is the initial configuration of the occlusal contact. This includes whether the initial contact was a one‐point or multiple‐point contact, or if it was located on inclined planes, which could potentially minimise lateral forces acting on the implant. These factors may significantly influence subsequent changes, warranting further investigation [37, 38].

Several factors should be considered when interpreting the findings of this review. Although the T‐Scan and Dental Prescale systems are widely used and validated for assessing occlusal force [31, 39], these computerised systems, particularly the T‐Scan, have inherent limitations in terms of reproducibility and measurement validity [40]. Such limitations can contribute to the variability observed across study outcomes. Moreover, the majority of the included studies involved small sample sizes and a limited number of implants, thereby reducing the overall strength of the evidence. Additionally, as most prostheses were confined to the molar region, the generalisability of the results to other implant sites is limited.

## 5 Conclusion

The reviewed studies demonstrate that implant‐supported crowns progressively adapt to the masticatory system, eventually matching or even exceeding the load borne by natural teeth. This adaptation is characterised by increased occlusal force, expanded contact areas and prolonged occlusion times. These findings highlight the importance of continuously monitoring the occlusal condition of implant‐supported fixed prostheses to ensure their long‐term success and functionality.

Additionally, the need for more precise and advanced measurement techniques is evident. Such innovations are essential to provide a better understanding of the long‐term changes in occlusion associated with implant‐supported crowns and effectively manage such conditions.

## Author Contributions

I.A., R.H.P., H.H., and N.Y. contributed to the study design, performed the experiment, analysed the data, and prepared the manuscript. I.A., R.H.P., H.H., T.K., T.C., and N.Y. performed the data curation. I.A., R.H.P., H.H., and N.Y. performed the formal analysis and interpretation. I.A., R.H.P., H.H., and T.K. prepared the original draft. P.R. and N.Y. substantially revised the manuscript. All authors read and approved the final manuscript.

## Conflicts of Interest

The authors declare no conflicts of interest.

## Peer Review

The peer review history for this article is available at https://www.webofscience.com/api/gateway/wos/peer‐review/10.1111/joor.70015.

## Supporting information


Data S1

## Data Availability

The data that support the findings of this study are available from the corresponding author upon reasonable request.
