# Supplementary material for: Occlusal Contact Changes in Implant‐Supported Fixed Prostheses: A Systematic Review
Source: J Oral Rehabil. 2025 Jul 24;52(11):2185–94. doi: 10.1111/joor.70015 (PMC12516001; doi:10.1111/joor.70015)
Supplement: Supplementary file 1 — Data S1 [file JOOR-52-2185-s001.docx]

|  | Supplementary 1: Search keys |  |
| --- | --- | --- |
| **Pubmed** |  |  |
| **#** | **Search terms** |  |
| #1 | ("implant-supported single crown*" [Text Word] OR "implant-supported crown*" [Text Word] OR "single implant crown*" [Text Word] OR "implant crown" [Text Word] OR "implant supported" [Text Word] OR "implant-supported fixed prosthes*" [Text Word] OR "Implant Restoration" [Text Word] OR "screw- retained" [Text Word] OR "dental implant" [Text Word]) OR ("Dental Implants*" [MeSH Terms] OR "Prosthesis Implantation" [MeSH Terms] OR "Dental Implants, Single-Tooth*" [MeSH Terms] OR "Dental Prosthesis, Implant-Supported" [MeSH Terms]) |  |
| #2 | ("changes over time" [Text Word] OR "Longitudinal changes" [Text Word] OR "Time-dependent changes" [Text Word] OR "Long-term" [Text Word] OR "?-year" [Text Word] OR "?-month" [Text Word]) OR ("Follow-Up Studies" [MeSH Terms] OR "Prospective Studies" [MeSH Terms]) |  |
|  |  |  |
| #3 | ("occlusal contact" [Text Word] OR "occlusal contact area" [Text Word] OR "occlusal scheme" [Text Word] OR "T-Scan" [Text Word] OR "Occlufast" [Text Word] OR "Occlusal variation" [Text Word] OR "Occlusal distribution" [Text Word] OR "occlusal surface" [Text Word] OR "occlusal area" [Text Word]) OR ("dental occlusion" [MeSH Terms]) |  |
| Search combination | #1 AND #2 AND #3 |  |
|  |  |  |
| **Scopus** |  |  |
| **#** | **Search terms** |  |
| #1 | ( TITLE-ABS-KEY ( "implant-supported single crown*" ) OR TITLE-ABS-KEY ( "implant-supported crown*" ) OR TITLE-ABS-KEY ( "single implant crown*" ) OR TITLE-ABS-KEY ( "implant crown" ) OR TITLE-ABS-KEY ( "implant supported" ) OR TITLE-ABS-KEY ( "implant-supported fixed prosthes*" ) OR TITLE-ABS-KEY ( "Implant Restoration" ) OR TITLE-ABS-KEY ( "screw- retained" ) OR TITLE-ABS-KEY ( "Dental Implant*" ) OR TITLE-ABS-KEY ( "Prosthesis Implantation" ) ) |  |
| #2 | ( TITLE-ABS-KEY ( "changes over time" ) OR TITLE-ABS-KEY ( "Longitudinal changes" ) OR TITLE-ABS-KEY ( "Time-dependent changes" ) OR TITLE-ABS-KEY ( "Long-term" ) OR TITLE-ABS-KEY ( "?-year" ) OR TITLE-ABS-KEY ( "?-month" ) ) |  |
|  |  |  |
| #3 | ( TITLE-ABS-KEY ( "occlusal contact" ) OR TITLE-ABS-KEY ( "occlusal contact area" ) OR TITLE-ABS-KEY ( "occlusal scheme" ) OR TITLE-ABS-KEY ( "T-Scan" ) OR TITLE-ABS-KEY ( "Occlufast" ) OR TITLE-ABS-KEY ( "Occlusal variation" ) OR TITLE-ABS-KEY ( "Occlusal distribution" ) OR TITLE-ABS-KEY ( "occlusal surface" ) OR TITLE-ABS-KEY ( "occlusal area" ) OR TITLE-ABS-KEY ( "dental occlusion" ) ) |  |
| Search combination | #1 AND #2 AND #3 |  |
|  |  |  |
|  |  |  |
|  |  |  |
| **Web of Science** |  |  |
| **#** | **Search terms** |  |
| #1 | ((((((((TS=("implant-supported single crown")) OR TS=("implant-supported crown")) OR TS=("single implant crown")) OR TS=("implant crown")) OR TS=("implant supported")) OR TS=("implant-supported fixed prostheses")) OR TS=("Implant Restoration*")) OR TS=("screw-retained")) OR TS=("Dental Implant*") |  |
| #2 | (((((TS=("changes over time")) OR TS=("Longitudinal changes")) OR TS=("Time-dependent changes")) OR TS=("Long-term")) OR TS=("?-year")) OR TS=("?-month") |  |
|  |  |  |
| #3 | ((((((((TS=("implant-supported single crown")) OR TS=("implant-supported crown")) OR TS=("single implant crown")) OR TS=("implant crown")) OR TS=("implant supported")) OR TS=("implant-supported fixed prostheses")) OR TS=("Implant Restoration*")) OR TS=("screw-retained")) OR TS=("Dental Implant*") |  |
| Search combination | #1 AND #2 AND #3 |  |
|  |  |  |
| **Cochrane via Ovid** |  |  |
| **#** | **Search terms** |  |
| #1 | ("implant-supported single crown*" or "implant-supported crown*" or "single implant crown*" or "implant supported" or "implant-supported fixed prostheses" or "Implant Restoration" or "screw- retained" or "dental implant").mp. [mp=ti, ab, tx, ct, mi, ot, mx, nt, hw, tc, id, tm, mf, kw, fx, sh, bt, nm, kf, ox, px, rx, ui, sy, ux] |  |
| #2 | ("changes over time" or "Longitudinal changes" or "Time-dependent" or "Long-term" or "?-year" or "?-month").mp. [mp=ti, ab, tx, ct, mi, ot, mx, nt, hw, tc, id, tm, mf, kw, fx, sh, bt, nm, kf, ox, px, rx, ui, sy, ux] |  |
|  |  |  |
| #3 | ("occlusal contact" or "occlusal contact area" or "occlusal scheme" or "T-Scan" or "Occlufast" or "Occlusal variation" or "Occlusal distribution" or "occlusal surface" or "occlusal area" or "dental occlusion").mp. [mp=ti, ed, ot, ab, tx, ct, sh, mi, mx, nt, hw, kw, tc, id, tm, mf, fx, bt, nm, kf, ox, px, rx, an, ui, sy, ds, on, ux] |  |
| Search combination | #1 AND #2 AND #3 |  |
